# Predictive Potential of Flux Balance Analysis of *Saccharomyces cerevisiae* Using as Optimization Function Combinations of Cell Compartmental Objectives

**DOI:** 10.1371/journal.pone.0043006

**Published:** 2012-08-09

**Authors:** Carlos Eduardo García Sánchez, César Augusto Vargas García, Rodrigo Gonzalo Torres Sáez

**Affiliations:** 1 Escuela de Ingeniería Química, Grupo de Investigación en Bioquímica y Microbiología, Universidad Industrial de Santander, Bucaramanga, Santander, Colombia; 2 Escuela de Ingeniería de Sistemas e Informática, Grupo de Investigación en Ingeniería Biomédica, Universidad Industrial de Santander, Bucaramanga, Santander, Colombia; 3 Escuela de Química, Grupo de Investigación en Bioquímica y Microbiología, Universidad Industrial de Santander, Bucaramanga, Santander, Colombia; Semmelweis University, Hungary

## Abstract

**Background:**

The main objective of flux balance analysis (FBA) is to obtain quantitative predictions of metabolic fluxes of an organism, and it is necessary to use an appropriate objective function to guarantee a good estimation of those fluxes.

**Methodology:**

In this study, the predictive performance of FBA was evaluated, using objective functions arising from the linear combination of different cellular objectives. This approach is most suitable for eukaryotic cells, owing to their multiplicity of cellular compartments. For this reason, *Saccharomyces cerevisiae* was used as model organism, and its metabolic network was represented using the genome-scale metabolic model iMM904. As the objective was to evaluate the predictive performance from the FBA using the kind of objective function previously described, substrate uptake and oxygen consumption were the only input data used for the FBA. Experimental information about microbial growth and exchange of metabolites with the environment was used to assess the quality of the predictions.

**Conclusions:**

The quality of the predictions obtained with the FBA depends greatly on the knowledge of the oxygen uptake rate. For the most of studied classifications, the best predictions were obtained with “maximization of growth”, and with some combinations that include this objective. However, in the case of exponential growth with unknown oxygen exchange flux, the objective function “maximization of growth, plus minimization of NADH production in cytosol, plus minimization of NAD(P)H consumption in mitochondrion” gave much more accurate estimations of fluxes than the obtained with any other objective function explored in this study.

## Introduction

Gradual development on genetic manipulation techniques has opened great possibilities for alteration of microorganisms for different purposes. These approaches have ranged from improvements and developments in the production of several metabolites, to multiple biochemical and microbiological investigations [1]. Since early developments in this field, the need for global analysis of cellular systems was evident, because interaction between cellular components does not allow cell functions to be explained simply by characterizing the components comprised in it [2].

This environment led to the emergence of metabolic engineering, which is a combination of systematic analysis from different cellular networks (metabolic, signaling, etc.) with molecular biology techniques to improve cellular properties through rational design and the implementation of genetic modifications [1]. Among the areas studied by metabolic engineering, one of the most relevant fields is searching for techniques to quantitatively predict the metabolic behavior of microorganisms under different conditions. In this category, the most widely used mathematical modeling approach has been flux balance analysis (FBA) [3].

FBA is based on the assumption that evolutionary pressure has led to the redirection of cellular metabolic fluxes, seeking for an optimal distribution according to a certain cellular goal [4]. This assumption make it possible to solve (i.e. to find a flux distribution based on) the underdetermined system that results from a mass balance in steady state of the intracellular metabolites [3], shown in equation (1), transforming the issue into the optimization problem of the equation (2).
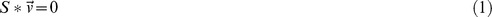


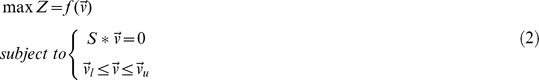



In equations (1) and (2), 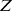 is the objective function that represents the cellular goal, 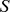
is the stoichiometric matrix, 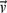 is the flux value vector, and 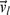 and 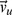 are the lower and upper bounds of the flux values, respectively. It is evident that the flux distribution estimated by the FBA depends on the objective function used, and therefore the chosen goal will have a direct impact on the quality of the predictions. It has been shown that, qualitatively, simulations carried out with FBA are consistent with experimental data [5], but in many cases, quantitative predictions are not reliable.

To apply FBA as a predictive technique, it should be ensured that fluxes predicted clearly represent cell growth and exchange of metabolites by only using information related to the medium in which cells are growing as input data. For this aim, it is necessary to have metabolic models of higher quality, to improve the available knowledge about the restrictions on the metabolic fluxes, and to obtain objective functions that represent in a better way the biological goals.

In most analysis, maximization of biomass production has been assumed as the most appropriate objective function (e.g. [6]–[12]). Recently, this objective function has been reviewed [13]. However, it has been found that growth-based optimization may not occur in all substrates [9], and that in some cases other objective functions perform better adjustments (e.g. [14]–[16]).

The problem of creating objective functions from experimental data has already been addressed; for example, finding the coefficients of importance (*CoIs*), representing the consistency of the hypothesis that a given flux is maximized by the organism as part of its cellular objective [17], or with the BOSS method, in which an objective function is generated from the stoichiometric network, together with constraints over the fluxes and a set of experimental data [18]. However, the objective functions obtained with these approaches are highly dependent on particular data sets, and cannot always be interpreted from a physiological point of view.

This work was aimed to determine whose FBA objective functions, composed of linear combination of objectives that represents targets of the cell compartments, allow better predictions of the metabolic fluxes. There are many objective functions previously proposed that are included in the linear combinations studied. Errors in predictions of both cell growth and exchange of metabolites (i.e. excretion) were evaluated quantitatively. The methodology presented here is most suitable for eukaryotic organisms, because prokaryotes do not possess multiple organelles. Therefore, as *Saccharomyces cerevisiae* is generally used as the eukaryotic model organism, experimental data and a metabolic model of this microorganism were used for the calculations [19]. While most FBA performance evaluations have been done using moderate size stoichiometric models, this study used a genome-scale model of the metabolism of *S. cerevisiae*, which led to the use of much of the information available about its metabolism. The experimental data sets were classified in various categories, according to growth and environmental conditions.

To sum up, the performance of different FBA objective functions (composed by linear combination of different compartmental objectives) was assessed, using a genome-scale metabolic network as metabolic model, and experimental data to determine the quality of every objective function. The objective functions evaluated can be represented by equation (3).
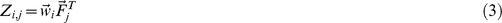




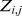
 are the FBA objective functions tested in the study, 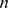 is the number of cellular compartments considered, 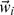 is an (1×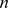) row vector of relative weighting, and 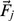 is an (1×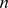) row vector whose *o*-th element correspond to a possible objective of the *o*-th cellular compartment (the *T* superscript in equation (3) indicates transposition).

Errors in the estimations produced for the FBA when every 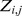 was used as objective function were evaluated, comparing exchange fluxes and biomass production predictions with experimental data. The tested combinations of compartmental objectives were ranked according to the absolute value of the error percentage in the prediction of the specific growth rate, when the combination of objectives is used as objective function in the FBA. This approach can be expressed as shown by equation (4), but instead of selecting only one combination of objectives (i.e. solving the outer optimization problem) for every category, the five best combinations of objectives for every category (of environmental/growth conditions) were analyzed; the errors in the estimated values obtained with those five best objective functions in the FBA were compared with the errors found using the most popular objective function (i.e. maximization of biomass production), and the errors found with an objective function that generally does not give good predictions (i.e. maximization of ATP production).
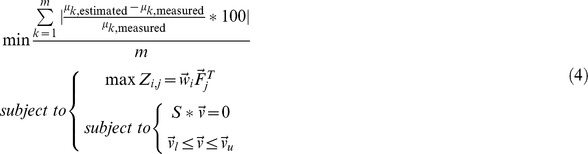



In equation (4), 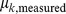 is the measured specific growth rate in the *k*-th experimental data set of the category, 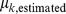 is the specific growth rate predicted by the internal optimization problem (i.e. a FBA done using 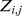 as objective function) for the *k*-th data set, and 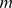 is the number of experimental data sets that belongs to the category (of environmental/growth conditions) analyzed.

## Methods

Multiple data sets of *S. cerevisiae* growth experiments were obtained from the literature, forming a wide range of conditions for the estimations so that the conclusions obtained would be as versatile as possible. Subsequently, selected data sets went under a carbon balance analysis, to avoid considering experimental data with too much error, or too much uncertainty in the measurement. We excluded data sets with errors greater than 10% in the overall mass balance. **Table 1** shows the list of experimental data sets taken into account in this study, and some important experimental conditions from the experiments carried out in the selected data sets from every listed source. Table S1 presents a more detailed description of the experimental data sets used.

**Table 1:** Experimental data used in this study. Characteristics, quantity and source of the experimental data used for the numerical evaluation of the performance of the FBA predictions, using combinations of cellular objectives as objective function.

Substrate	Culture conditions	Exchange fluxes measured or adapted	Amount of experimental data	Reference
Glucose	Continuous culture, respiro-fermentative	Ethanol, glycerol, acetate, succinate, acetaldehyde, pyruvate	1	[**20**]
Glucose	Continuous culture, anaerobic	Ethanol, glycerol, acetate, succinate, pyruvate, carbon dioxide	4	[**21**]
Glucose	Continuous culture, aerobic	Ethanol, glycerol, acetate, succinate, pyruvate	1	[**22**]
Glucose, maltose, ethanol, acetate	Continuous culture, aerobic	Oxygen, carbon dioxide	4	[**23**]
Glucose	Continuous culture, aerobic and respiro-fermentative	Ethanol, glycerol, acetate, carbon dioxide	3	[**24**]
Glucose plus ethanol	Continuous culture, aerobic	Oxygen, carbon dioxide	1	[**25**]
Galactose	Continuous culture, aerobic	Ethanol, glycerol, acetate, pyruvate, carbon dioxide	1	[**26**]
Glucose	Continuous culture, respiro-fermentative and anaerobic	Ethanol, glycerol, acetate, oxygen	8	[**27**]
Glucose	Continuous culture, respiro-fermentative and anaerobic	Ethanol, glycerol, acetate, oxygen	4	[**28**]
Glucose	Exponential growth in batch culture, respiro-fermentative	Ethanol, glycerol, acetate, carbon dioxide	8	[**29**]
Glucose	Continuous culture, aerobic	Ethanol, glycerol, acetate, pyruvate, oxygen, carbon dioxide	10	[**30**]

A genome-scale compartmentalized model, the iMM904 model, was selected as the metabolic model of *S. cerevisiae*. This model consists of 1577 reactions (including transport reactions) and 1228 metabolites, distributed in eight locations: extracellular space, cytosol, mitochondria, peroxisome, nucleus, endoplasmic reticulum, Golgi apparatus and vacuole [31]. As it can be seen, the model has seven cellular compartments that could be considered as contributors to the global cellular objective. However, the number of reactions in the compartments has different orders of magnitude, so it was decided to consider the five compartments with higher number of reactions as possible contributors to the cellular objective function. The five compartments that accomplish this condition are cytosol, mitochondria, nucleus, endoplasmic reticulum and peroxisome. The other two compartments, vacuole and Golgi apparatus, only contain three and six reactions, respectively.

Subsequently, some preliminary tests were addressed to explore the influence of the objective functions from the compartments in the modeling. For each one of these five compartments, a number of potential objectives were selected, and their linear combinations would compose the objective functions studied. These preliminary tests showed that the maximization of growth was one of the functions that had a greater impact on the quality of the predictions, and that potential objectives from the compartments “nucleus” and “endoplasmic reticulum” did not contribute adequately to the quality of the estimations, possibly because of the insufficient connectivity of metabolites within these compartments in the model [32]. Therefore, those two compartments were discarded in the modeling of this study, and the objective functions related to the biomass were moved into a virtual compartment, named “global”. **Table 2** shows the different possible objectives considered for every compartment studied; those objectives are related mainly to production and consumption of reductive power and ATP, owing to the outstanding importance of these cofactors in explaining the cellular behavior. Other objectives considered are associated with biomass production, because the relevance of this objective in the FBA modeling is well established, and with production of diverse secondary metabolites.

**Table 2:** Compartmental objectives considered in the study. List of the possible compartmental objectives evaluated in this study. All the combinations of these objectives create the objective functions whose predictive potential was evaluated.

Global	Cytosol	Mitochondria	Peroxisome
max (biomass production)	max (NAD(P)H production)	max (ATP production)	max (fatty acids production)
min (biomass production)	max (NAD(P)H consumption)	max (NAD(P)H production)	max (ATP consumption)
	min (NADH production)	min (NAD(P)H consumption)	min (ATP consumption)
	min (NAD(P)H consumption)	max (transport of ATP towards cytosol)	max (NAD(P)H production)
	min (NAD(P)H production)		min (NAD(P)H consumption)
	max (ATP production)		
	min (ATP consumption)		
	min (acetate production)		
	min (CO_2_ production)		
	min (ethanol production)		
	min (succinate production)		
	min (glycerol production)		
	max (acetate production)		
	max (CO_2_ production)		
	max (ethanol production)		
	max (succinate production)		
	max (glycerol production)		

The objective functions evaluated in this study consisted of linear combinations from the objectives of the compartments, as was established in equation (3). The evaluated relative weightings represent a big amount of different ratios (e.g., 1∶1∶1∶1, 1∶2∶3∶4, 1∶1∶1∶4, 1∶1∶1∶8, etc); vectors 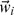
with elements equal to zero were also included allowing the evaluation of objective functions composed by subsets of compartmental objectives, and even each possible objective independently. Table S2 shows the complete list of the relative weighting vectors used in this study. Many previously evaluated objective functions (e.g. [6], [15], [16]) are also represented, including maximization of biomass production {*Z* =  (max biomass production)_glb_}, minimization of reductive power {*Z* =  (min NAD(P)H production)_cyt_+(min NAD(P)H consumption)_mit_+(min NAD(P)H consumption)_per_}, maximization of ATP production rate {*Z* =  (max ATP production)_cyt_+(max ATP production)_mit_}, and minimization of production of NADH {*Z* =  (min NADH production)_cyt_}.

This study was aimed to compare predictive potential from the different objective functions formed by the combinations of the compartment objectives, as mentioned above. Therefore, only the flux values corresponding to substrate uptake and (if available) exchange of oxygen, were used as input data in each FBA executed. Values of biomass production and other fluxes determined experimentally in the data sets were used to quantify the predictive potential of the diverse objective functions. Errors in the estimation of biomass production for the *k*-th data set were calculated as percentage of relative error and were named “*Biomass error*”, as displayed in equation (5).
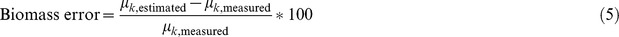



The sign of *Biomass error* indicates if the prediction overestimates the growth (positive sign) or underestimates it (negative sign), and this was utilized in the analysis of the different objective functions. However, for rating the objective functions, the absolute value of this kind of error was used, as was shown in equation (4).

On the other hand, errors in the prediction of the (experimentally known) exchange fluxes were calculated as the Euclidean distance between the experimental values of the exchange fluxes and the FBA estimated values; this kind of error was named “*Exchange fluxes error*”, and its calculation is presented in equation (6).
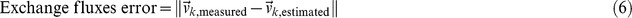



In equation (6), 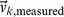 is the vector of measured values of the exchange fluxes experimentally determined in the *k*-th data set, and 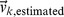 is the vector of the estimated values of the same exchange fluxes.

The simulations and comparisons were made with Matlab® (The Mathworks, Inc.) software and the COBRA toolbox package [33]. The linear programming solver used was the glpk free solver, with glpkmex link as the interface with Matlab.

## Results

The experimental data sets were classified into different categories to explore the relationship between environmental conditions and the objective functions that best model cell behavior, as well as the relationship between cell growth and the better objective functions in FBA. The categories utilized to classify the data sets were dependent on the availability of oxygen in the media, on the specific growth rate, and on the type of substrate.

The results obtained in the simulations for every category were ordered according to the accuracy in estimating the growth rate, but both classes of error calculated (i.e. *Biomass error* and *Exchange fluxes error*) were considered in the analyses. The errors are shown in the figures using box-and-whisker plots, with blue color for *Biomass error* and green color for *Exchange fluxes error*. In general, the objective functions that performed better estimations with respect to the prediction of microbial growth had also errors in the estimation of the exchange fluxes that were among the lowest ones.

The analysis carried out consisted in identifying trends among the best functions, rather than limiting the analysis to the objective function that gives the best performance in FBA. It was better to do it that way because of the existence of random error during experiments, the diversity of experimental techniques used for obtaining data in this work, the small difference in errors obtained with several of the functions and the fact that values calculated using FBA represent a “average cell” in pseudo-stable state [34]. The figures show the errors in the predictions of the best five objective functions (i.e. the five combinations of compartmental objectives which cause better growth predictions), and the errors in the predictions of two global objectives: “maximization of growth” (being the most used FBA objective until now) and “maximization of ATP production” (this objective always predicts a growth rate of zero, because the fluxes are oriented towards other pathways rather than biomass production), for comparison in every category studied.

The simulations showed that the possible peroxisomal objectives did not play any role on the predictions obtained with the FBA, since its contribution in every case was null or not significant.

Because of this, the figures and results do not mention any peroxisomal objective. Another unexpected phenomenon found was that all the different estimations obtained could be represented using only ones and zeros as relative weightings (i.e. all the predictions obtained using relative weightings that include values different to –exclusively– ones and zeros were equal to the obtained with some relative weighting composed only by ones and zeros). Therefore, the results in the figures do not show relative weighting values because all of them are ones and zeros (a weight that is zero means that none of the objectives from this compartment is active).

### Predictive potential concerning availability of oxygen

**Figure 1:**
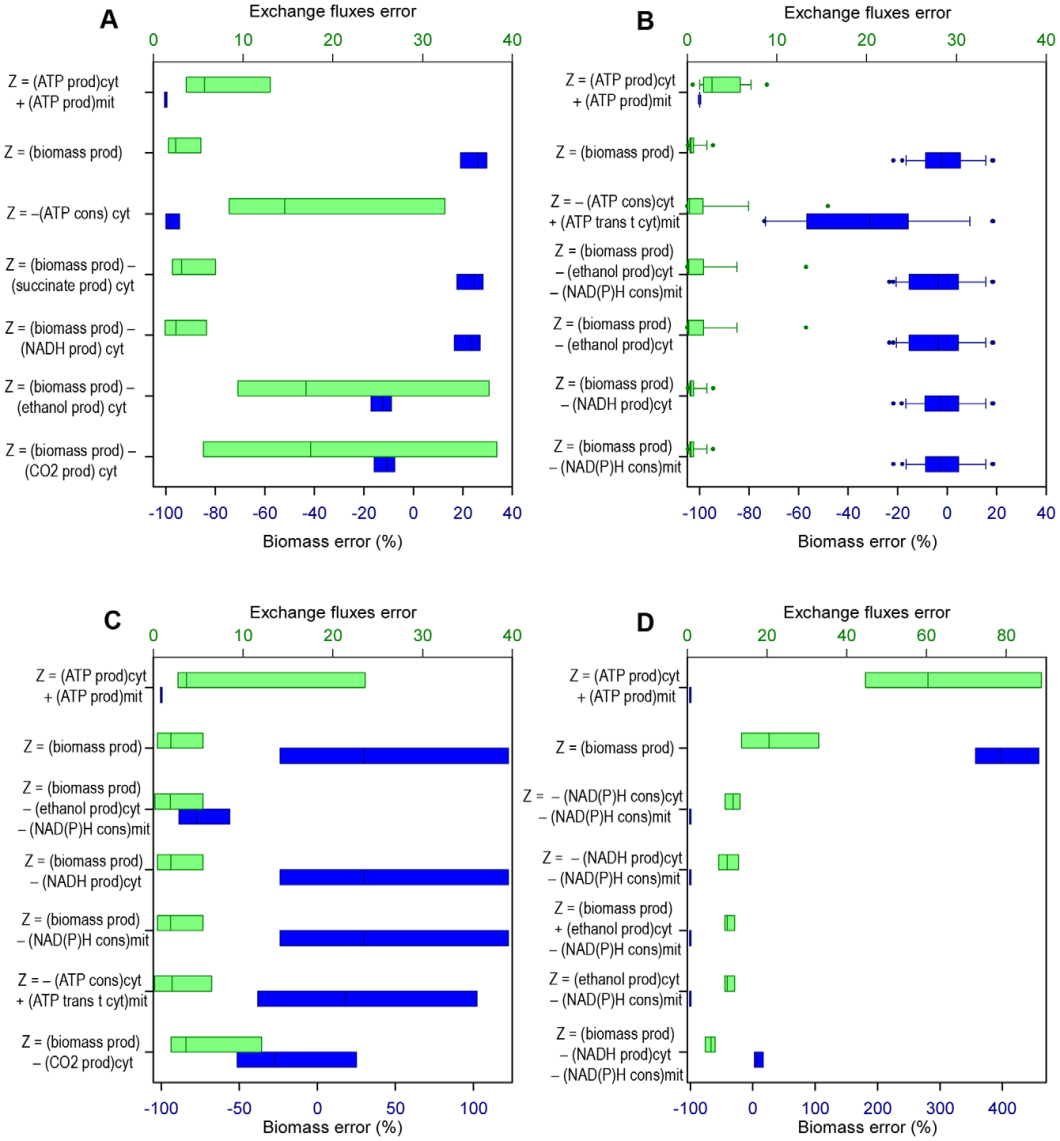
Best objective functions for FBA, regarding presence of oxygen in the medium. Errors of the FBA estimations using the five best compartmentalized objective functions ***Z*** (for every category), and “max biomass production” and “max ATP production” functions. The compartmental objectives that correspond to minimizations have negative sign, so ***Z*** is always maximized. *Biomass error*: error percentage in the estimation of the specific growth rate (blue boxes). *Exchange fluxes error*: Euclidean distance between the estimated values and the experimental values of the known exchange fluxes (green boxes). **A**: Anaerobic growth. **B**: Aerobic growth with known flux exchange of oxygen. **C**: Aerobic growth with unknown oxygen uptake. **D**: Aerobic growth with unknown oxygen uptake, in batch experiments (exponential phase). *prod.*: production; *cons.*: consumption; *cyt*: cytosolic; *mit*: mitochondrial; *trans t cyt*: transport towards cytosol.

The experimental data [20]–[30] were classified according to the availability of oxygen in the cell culture, categorizing those in anaerobic growth in continuous culture (six of the data sets utilized belong to this category), aerobic growth with known oxygen uptake rate in continuous culture (25 data sets), aerobic growth with unknown oxygen uptake rate in continuous culture (six data sets), and aerobic exponential growth in a batch reactor with unknown oxygen uptake rate (eight data sets). This subdivision from the aerobic growth cases was made because there is a huge difference in the accuracy of the FBA predictions between these cases. When oxygen uptake is unknown, the oxygen flux in the FBA restrictions was bounded by a lower limit of zero, while the upper limit was a value higher than the experimental oxygen uptake measured in experiments with high growth rates, and this caused that accuracy of the estimations was more limited. This election of the upper limit in the oxygen flux value was done to compare the performance of the different objective functions when there is lack of information about this flux. **Figure 1** shows the predictions errors from the five best objective functions, compared to the errors obtained with maximization of growth and maximization of ATP production as objective functions, for each one of the categories mentioned at the start of this section.

In the case of anaerobic growth (**Figure 1A**), the best predictions were obtained with “maximization of growth”, “maximization of growth plus minimization of NADH production in cytosol” and “maximization of growth plus minimization of succinate production in cytosol”. Based on the data employed, these three objectives led to an overestimation of the growth rate of about 25%, and a low error in the exchange fluxes' prediction. The other two interesting objectives were “maximization of growth plus minimization of ethanol production in cytosol” and “maximization of growth plus minimization of CO_2_ production in cytosol” These two objectives led to a slightly better prediction of growth rate (less than 20%), but underestimating it. Anyway, the other fluxes' estimations for those two functions are much poorer, so these combinations are not good objectives. The other possible combinations evaluated led to significantly worst predictions. For example, the fifth best objective function (“minimization of ATP consumption in cytosol”) had a *Biomass error* of almost 100%.

In the second case (aerobic growth with known oxygen uptake rate (**Figure 1B**)) “maximization of growth” and “maximization of growth plus minimization of NADH production in cytosol” were again between the best objective functions, and “maximization of growth plus minimization of NAD(P)H consumption in mitochondrion” achieved the same prediction error. Additionally, other two objective functions presented a comparable prediction level, and the rest of the combinations of compartmental objectives led to estimations of much lower quality.

The third category (aerobic growth with unknown oxygen uptake rate (**Figure 1C**)) shows some functions with a precision comparable to that of “maximization of growth” again, and between those functions we can also find “maximization of growth plus minimization of NADH production in cytosol”. It is evident that the lack of knowledge about the oxygen uptake rate causes a tendency to considerably overestimate the growth rate, exceeding 100% of error in some cases, for the combinations of objective functions having “maximization of biomass” between its elements. Only one of the best functions, “maximization of growth plus minimization of CO_2_ production in cytosol”, tends to underestimate the growth instead of overestimating it, showing only a slightly worst exchange fluxes' prediction. But in general, the predictions are worst than the ones obtained when the oxygen uptake rate is known (it is clear that in the anaerobic case the oxygen uptake is also known, being equal to zero).

The trends were totally different in the fourth case (aerobic exponential growth in a batch reactor with unknown oxygen uptake rate (**Figure 1D**)). “Maximization of growth” now causes a *Biomass error* higher than 300% in all studied data sets. Quantitatively, the best predictions were obtained with functions that predict no growth (i.e. underestimating the growth by 100%), except for one function, that really stands out among all: the combination “maximization of growth plus minimization of NADH production in cytosol plus minimization of NAD(P)H consumption in mitochondrion”; which permits to obtain predictions with *Biomass error* of less than 20%, and *Exchange fluxes error* lower than 10. **Figure 1D** clearly shows the big difference in the predictions obtained with the previously mentioned combination of objectives for the data sets evaluated, which seem to be a very attractive option for modeling exponential growth phase of *S. cerevisiae*. This indicates that the addition of the objectives “minimization of NADH production in cytosol plus minimization of NAD(P)H consumption in mitochondrion” to the “maximization of biomass” objective causes that the objective function models the real oxygen uptake rate in an outstanding way, in cases where the growth rate is high (exponential growth), despite the lack of accurate information about the oxygen exchange flux.

### Predictive potential concerning growth rate, in aerobic growth with known oxygen flux

**Figure 2:**
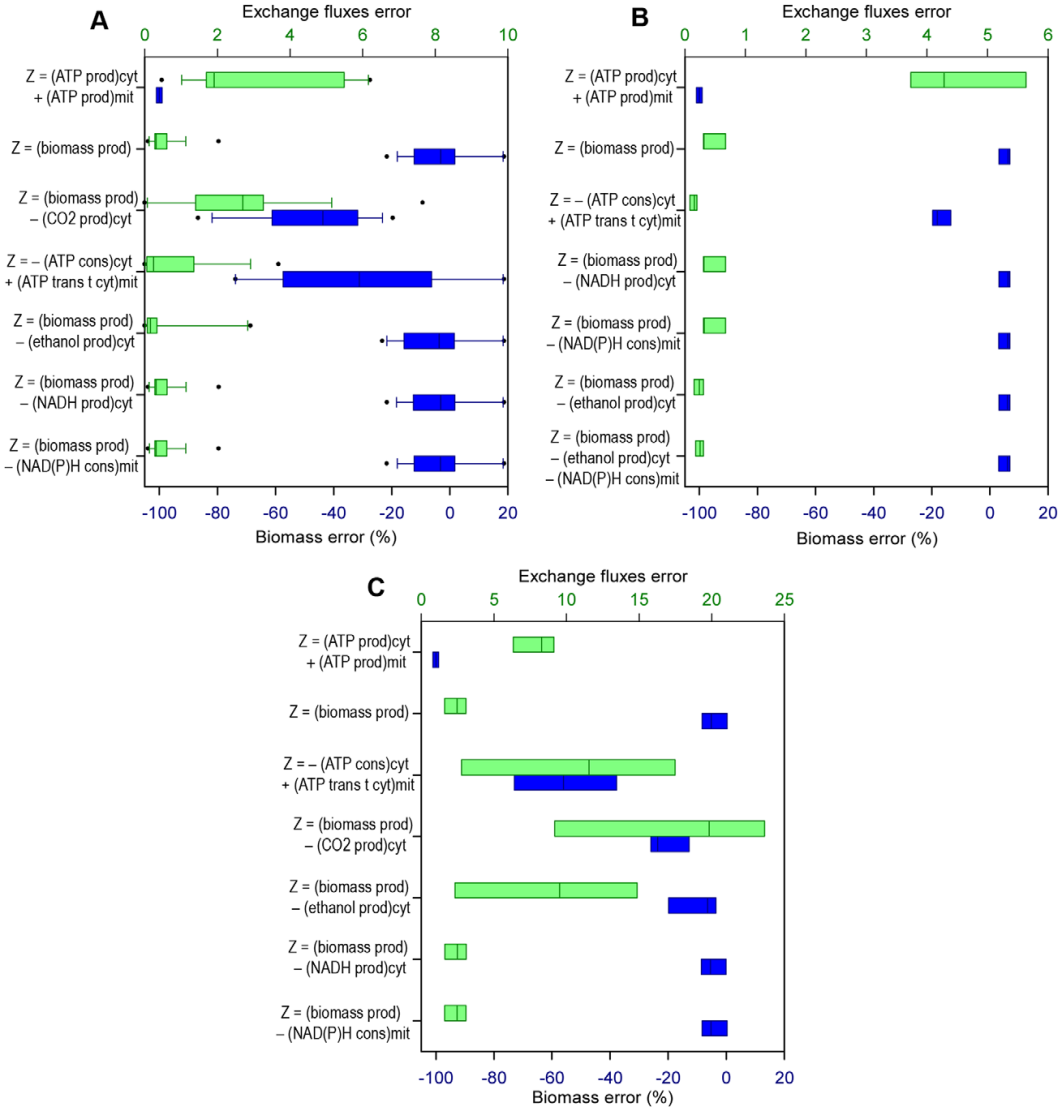
Best objective functions for FBA, regarding specific growth rate, knowing the oxygen uptake rate. Errors of the FBA estimations using the five best compartmentalized objective functions ***Z*** (for every clasification), and “max biomass production” and “max ATP production” functions. The compartmental objectives that correspond to minimizations have negative sign, so ***Z*** is always maximized. *Biomass error*: error percentage in the estimation of the specific growth rate (blue boxes). *Exchange fluxes error*: Euclidean distance between the estimated values and the experimental values of the known exchange fluxes (green boxes). **A**: Growth rate less than or equal to 0.15 h^−1^. **B**: Growth rate higher than 0.15 h^−1^ and lower than or equal to 0.28 h^−1^. **C**: Growth rate higher than 0.28 h^−1^. *prod.*: production; *cons.*: consumption; *cyt*: cytosolic; *mit*: mitochondrial; *trans t cyt*: transport towards cytosol.

The data with measured oxygen consumption came from chemostat experiments with different dilution rates, so it was decided to explore the performance of the functions according to growth rates. The data was classified into three subgroups: growth rate less than or equal to 0.15 h^−1^ (19 of the data sets utilized belong to this category), growth rate higher than 0.15 h^−1^ and lower than or equal to 0.28 h^−1^ (three data sets), and growth rate higher than 0.28 h^−1^ (three data sets). **Figure 2** shows the five objective functions that cause better FBA predictions in each one of these categories, and the two objectives used for comparison.

With the three ranges of growth rates, the tendency was that the best predictions were given for “maximization of growth”, and a couple of combinations including it (between them “maximization of growth plus minimization of NADH production in cytosol” and “maximization of growth plus minimization of NAD(P)H consumption in mitochondrion” in the three cases). The only case in which a better prediction is found among these three categories is in medium growth rates (i.e. growth rates between 0.15 h^−1^ and 0.28 h^−1^, see Figure 2B), where “maximization of growth plus minimization of ethanol production in cytosol” and “maximization of growth plus minimization of ethanol production in cytosol plus minimization of NAD(P)H consumption in mitochondrion” led to lower values of *Exchange fluxes error*, with an equal *Biomass error*.

### Predictive potential concerning type of substrate, in aerobic growth with known oxygen flux

**Figure 3:**
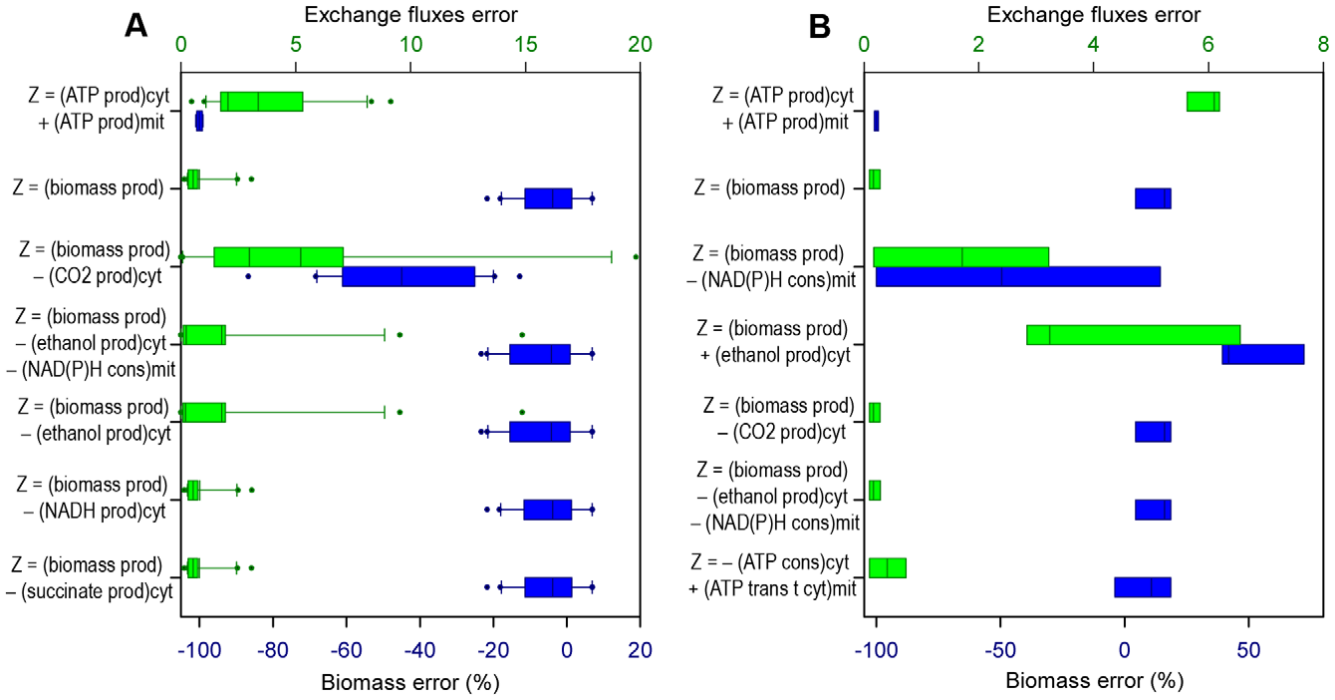
Best objective functions for FBA, regarding substrate type, knowing the oxygen uptake rate. Errors of the FBA estimations using the five best compartmentalized objective functions ***Z*** (for every case), and “max biomass production” and “max ATP production” functions. The compartmental objectives that correspond to minimizations have negative sign, so ***Z*** is always maximized. *Biomass error*: error percentage in the estimation of the specific growth rate (blue boxes). *Exchange fluxes error*: Euclidean distance between the estimated values and the experimental values of the known exchange fluxes (green boxes). **A**: Experiments with glucose as substrate. **B**: Experiments with substrates other than glucose. *prod.*: production; *cons.*: consumption; *cyt*: cytosolic; *mit*: mitochondrial; *trans t cyt*: transport towards cytosol.

Predictions of FBA with different objective functions in the cases in which the substrate was glucose (20 of the data sets utilized belong to this category) were compared with cases in which the substrate was not glucose (four data sets), because some previous studies have suggested that the best objective function in the application of FBA may depend on the type of substrate [9]. The combinations of objectives with better performance when the oxygen consumption is known and the substrate is glucose are shown in **Figure 3A**, and the best objectives for the experiments where oxygen flux was measured but substrate is not glucose, are depicted in **Figure 3B**.

In **Figure 3** it is shown that there is not much difference between the best objective functions in both cases. With or without glucose as substrate, the best objectives were “maximization of biomass production” and a couple of combinations containing the aforementioned objective. The only exception in this figure is the best function in the ‘substrate not glucose’ case: the combination “minimization of ATP consumption in cytosol plus maximization of ATP transport towards cytosol in mitochondrion”, with which slightly better growth prediction and almost equal exchange fluxes' prediction are obtained. Anyway, there was a lack of data with substrates other than glucose in the data sets used in this study, making this observation not very reliable. Nevertheless, this could be an interesting FBA objective to analyze by using a bigger amount of ‘substrate not glucose’ data.

## Discussion

Quality of the predictions obtained with FBA was dramatically dependent on the knowledge of the oxygen uptake rate. Previous studies used this flux to evaluate the quality of FBA predictions, but used the other exchange fluxes as input data [16]. As the predictions has a lower quality when the oxygen uptake rate is unknown, lack of information about oxygen flux is a big problem for using FBA as a predictive technique in aerobic cases, because it is very difficult to predict the exact value of oxygen uptake rate for a determined medium condition. In any case, for certain experimental conditions, lower and upper bounds of oxygen uptake rate can be determined. In consequence, restrictions of oxygen uptake can be tightened and therefore predictions of FBA can be remarkably improved.

For the most of studied classifications, the best predictions were obtained with “maximization of growth”, and other combinations that included this previous objective. This result agrees with previous studies that supported the use of maximization of biomass production as objective function for FBA (e.g. [6], [9], [18]). “Minimization of NADH production in cytosol” and “minimization of NAD(P)H consumption in mitochondrion” were two objectives that constantly showed up in combination with “maximization of growth” in the best objective functions for every classification. These two objectives did not modify greatly the predictions obtained by “maximization of growth”, but change slightly the estimations obtained. This biochemical condition has been observed in *S. cerevisiae*, that strives to minimize the excess NADH formation under anaerobic conditions [35]. The biomass yield was positively correlated with the net amount of NADH reoxidized in respiration and glycerol formation, indicating that the turnover of excess NADH from biosynthesis is an important factor influencing the biomass yield under oxygen-limiting conditions [36]. Minimization of redox potential had already showed good results as FBA objective function for *Escherichia coli* [16], so its relevance is not something new.

The case of exponential aerobic growth with unknown oxygen flux is the only exception to the tendency mentioned in the preceding paragraph. A previous study had already indicated that the best objective can be different depending on if the cells are growing in a batch or a continuous culture [15]. In the present study, the combination “maximization of growth plus minimization of NADH production in cytosol plus minimization of NAD(P)H consumption in mitochondrion” gives estimates of fluxes that are much more accurate than the given by any other objective function explored, when *S. cerevisiae* was growing in a batch culture. With the aforementioned combination, errors in the growth rate were lower than 20%, while all of the others objective functions explored gave predictions with -100% (no growth) o more than 300% of error in the estimation of growth. This is a really interesting result, and it seems valuable to explore the performance of this FBA objective function with a higher amount of exponential growth data sets.

An extensive redistribution of fluxes has been observed in anaerobic conditions compared to all the aerobic conditions [27]. In addition, under aerobic conditions *S. cerevisiae* regenerates NAD^+^ mainly through respiration. When limited oxygen availability restricts respiration, cells are forced to use other means for regeneration of NAD^+^, and mitochondrial NADH needs to be transported to the cytosol for re-oxidation. For the transport of NADH, mitochondrial alcohol dehydrogenase, encoded by *ADH3,* provides a probable redox shuttle [23], [37].

The results did not indicate that different objective functions allow a better performance of FBA depending on substrate type (glucose or not glucose), as proposed by a previous study [9]. However, there was an uncommon objective function with unexpectedly good performance when the substrate is not glucose (“minimization of ATP consumption in cytosol plus maximization of ATP transport towards cytosol in mitochondrion”). Anyway, the amount of experimental data in the both categories studied was very different, having too little data with substrates other than glucose. To obtain better conclusions regarding the type of substrate, it is necessary to perform the analysis using more experiments with different substrates, being able to assess the possible existence of differences according to the path where the metabolites are integrated into the metabolic network. Similarly, the assessment of the effect of other factors, such as pH or unfavorable osmotic pressure in the culture medium, requires more and better experiments.

## Supporting Information

Table S1 Detailed experimental data sets used in the study.
(DOC)

Table S2Relative weighting vectors 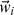 used for generate all the tested objective functions.(DOC)
