# Supplementary material for: Predictive Potential of Flux Balance Analysis of Saccharomyces cerevisiae Using as Optimization Function Combinations of Cell Compartmental Objectives
Source: PLoS One. 2012 Aug 9;7(8):e43006. doi: 10.1371/journal.pone.0043006 (PMC3415429; doi:10.1371/journal.pone.0043006)
Supplement: Table S1 — Detailed experimental data sets used in the study. (DOC) [file pone.0043006.s001.doc]

Table S1. Detailed experimental data sets used in the study.

| **Culture type** | **Substrate** | **Specific growth rate** $\mu$ | $v_{\text{substrate}}$ | $v_{\text{O}_{2}}$ | $v_{\text{ethanol}}$ | $v_{\text{C}O_{2}}$ | $v_{\text{glycerol}}$ | $v_{\text{acetate}}$ | $v_{\text{succinate}}$ | $v_{\text{acetaldehyde}}$ | $v_{\text{piruvate}}$ | **Reference** |
| --- | --- | --- | --- | --- | --- | --- | --- | --- | --- | --- | --- | --- |
| Continuous | Glucose | 0.1 | 3.002 | ND | 3.632 | ND | 0.2111 | 0.0078 | 0.0132 | 0.0048 | 0.0005 | **[20]** |
| Continuous | Glucose | 0.1 | 5.5550 | 0 | 8.2825 | 9.0658 | 0.0768 | 0.0333 | 0.0250 | ND | 0.0111 | **[21]** |
| Continuous | Glucose | 0.2 | 11.5022 | 0 | 17.1153 | 18.8406 | 2.0934 | 0.1035 | 0.0518 | ND | 0.046 | **[21]** |
| Continuous | Glucose | 0.3 | 17.3667 | 0 | 25.7374 | 27.8214 | 3.2997 | 0.3126 | 0.0521 | ND | 0.1042 | **[21]** |
| Continuous | Glucose | 0.4 | 23.6541 | 0 | 35.2682 | 37.0423 | 5.1566 | 0.7096 | 0.071 | ND | 0.1892 | **[21]** |
| Continuous | Glucose | 0.1 | 1.17 | ND | 0 | ND | 0 | 0 | 0 | ND | 0 | **[22]** |
| Continuous | Glucose | 0.1 | 1.15 | 2.74 | ND | 2.85 | ND | ND | ND | ND | ND | **[23]** |
| Continuous | Maltose | 0.1 | 0.61 | 3.05 | ND | 3.05 | ND | ND | ND | ND | ND | **[23]** |
| Continuous | Ethanol | 0.1 | 3.78 | 6.87 | ---**^*^** | 3.26 | ND | ND | ND | ND | ND | **[23]** |
| Continuous | Acetate | 0.1 | 5.89 | 7.40 | ND | 7.45 | ND | ---**^*^** | ND | ND | ND | **[23]** |
| Continuous | Glucose | 0.15 | 1.56 | ND | 0 | 3.6075 | 0.0325 | 0 | ND | ND | ND | **[24]** |
| Continuous | Glucose | 0.3 | 4.90 | ND | 2.4239 | 10.3213 | 0.2606 | 0.7037 | ND | ND | ND | **[24]** |
| Continuous | Glucose | 0.4 | 8.23 | ND | 8.8025 | 13.7405 | 0.5725 | 0.4294 | ND | ND | ND | **[24]** |
| Continuous | Glucose plus ethanol | 0.1 | 0.94 | 2.87 | 0.40 **^**^** | 2.64 | ND | ND | ND | ND | ND | **[25]** |
| Continuous | Galactose | 0.0970 | 1.06 | ND | 0 | 2.62 | 0 | 0 | ND | ND | 0.0005 | **[26]** |
| Continuous | Glucose | 0.1 | 1.28 | 2.70 | 0 | ND | 0 | 0 | ND | ND | ND | **[27]** |
| Continuous | Glucose | 0.1 | 1.36 | 2.50 | 0.07 | ND | 0 | 0 | ND | ND | ND | **[27]** |
| Continuous | Glucose | 0.1 | 1.28 | 2.50 | 0.10 | ND | 0 | 0 | ND | ND | ND | **[27]** |
| Continuous | Glucose | 0.1 | 1.97 | 1.70 | 1.56 | ND | 0 | 0 | ND | ND | ND | **[27]** |
| Continuous | Glucose | 0.1 | 2.13 | 1.70 | 2.00 | ND | 0 | 0 | ND | ND | ND | **[27]** |
| Continuous | Glucose | 0.1 | 2.78 | 1.20 | 2.91 | ND | 0 | 0 | ND | ND | ND | **[27]** |
| Continuous | Glucose | 0.1 | 6.30 | 0 | 9.05 | ND | 1.05 | 0 | ND | ND | ND | **[27]** |
| Continuous | Glucose | 0.1 | 6.58 | 0 | 9.47 | ND | 1.11 | 0 | ND | ND | ND | **[27]** |
| Continuous | Glucose | 0.1 | 1.10 | 2.70 | 0 | 2.60 | ND | ND | ND | ND | ND | **[28]** |
| Continuous | Glucose | 0.1 | 1.33 | 2.50 | 0.10 | 3.00 | ND | ND | ND | ND | ND | **[28]** |
| Continuous | Glucose | 0.1 | 1.90 | 1.70 | 1.60 | 3.70 | ND | ND | ND | ND | ND | **[28]** |
| Continuous | Glucose | 0.1 | 2.38 | 1.20 | 2.75 | 4.60 | ND | ND | ND | ND | ND | **[28]** |
| Batch (exponential growth) | Glucose | 0.4 | 20.2 | ND | 30.0 | 34.4 | 1.9 | 1.0 | ND | ND | ND | **[29]** |
| Batch (exponential growth) | Glucose | 0.16 | 7.2 | ND | 9.0 | 15.4 | 0.6 | 0.7 | ND | ND | ND | **[29]** |
| Batch (exponential growth) | Glucose | 0.4 | 19.9 | ND | 29.6 | 33.8 | 1.7 | 1.3 | ND | ND | ND | **[29]** |
| Batch (exponential growth) | Glucose | 0.36 | 18.4 | ND | 28.2 | 32.0 | 1.5 | 1.3 | ND | ND | ND | **[29]** |
| Batch (exponential growth) | Glucose | 0.21 | 12.3 | ND | 15.2 | 24.0 | 2.6 | 2.1 | ND | ND | ND | **[29]** |
| Batch (exponential growth) | Glucose | 0.17 | 10.2 | ND | 11.2 | 20.6 | 2.4 | 1.7 | ND | ND | ND | **[29]** |
| Batch (exponential growth) | Glucose | 0.33 | 15.1 | ND | 20.1 | 25.5 | 2.9 | 1.0 | ND | ND | ND | **[29]** |
| Batch (exponential growth) | Glucose | 0.23 | 12.2 | ND | 15.6 | 23.1 | 2.8 | 0.9 | ND | ND | ND | **[29]** |
| Continuous | Glucose | 0.025 | 0.3 | 0.8 | 0 | 0.8 | 0 | 0 | ND | ND | 0 | **[30]** |
| Continuous | Glucose | 0.05 | 0.6 | 1.3 | 0 | 1.4 | 0 | 0 | ND | ND | 0 | **[30]** |
| Continuous | Glucose | 0.1 | 1.1 | 2.5 | 0 | 2.7 | 0 | 0 | ND | ND | 0 | **[30]** |
| Continuous | Glucose | 0.15 | 1.7 | 3.9 | 0 | 4.2 | 0 | 0 | ND | ND | 0 | **[30]** |
| Continuous | Glucose | 0.2 | 2.3 | 5.3 | 0 | 5.7 | 0 | 0 | ND | ND | 0 | **[30]** |
| Continuous | Glucose | 0.25 | 2.8 | 7.0 | 0 | 7.5 | 0 | 0 | ND | ND | 0 | **[30]** |
| Continuous | Glucose | 0.28 | 3.4 | 7.4 | 0.11 | 8.0 | 0 | 0.08 | ND | ND | 0.01 | **[30]** |
| Continuous | Glucose | 0.3 | 4.5 | 6.1 | 2.3 | 8.8 | 0 | 0.41 | ND | ND | 0.01 | **[30]** |
| Continuous | Glucose | 0.35 | 8.6 | 5.1 | 9.5 | 14.9 | 0.05 | 0.62 | ND | ND | 0.03 | **[30]** |
| Continuous | Glucose | 0.4 | 11.1 | 3.7 | 13.9 | 18.9 | 0.15 | 0.6 | ND | ND | 0.05 | **[30]** |

Culture and substrate type, exchange fluxes measured, and source of the data sets used in the study. The specific growth rate is indicated in h^-1^, while the fluxes are shown in mmol/(gDW*h). The fluxes of the columns $v_{\text{substrate}}$and $v_{\text{O}_{2}}$correspond to uptake of metabolites, while the rest of the fluxes’ columns correspond to excretion of metabolites.

ND : No Data; in the reference, there was no data reported about this flux value.

^*^ : The flux value is not shown here, because the metabolite is the substrate and its flux appears in the $v_{\text{substrate}}$column.

^**^ : This exchange flux corresponds to ethanol uptake, not production.
