# Supplementary material for: Predictive Potential of Flux Balance Analysis of Saccharomyces cerevisiae Using as Optimization Function Combinations of Cell Compartmental Objectives
Source: PLoS One. 2012 Aug 9;7(8):e43006. doi: 10.1371/journal.pone.0043006 (PMC3415429; doi:10.1371/journal.pone.0043006)
Supplement: Table S2 — Relative weighting vectors used for generate all the tested objective functions. (DOC) [file pone.0043006.s002.doc]

**Table S2. Relative weighting vectors $\vec{w}_{i}$ used for generate all the tested objective functions**.

| $\vec{w}_{i}$ | | | |
| --- | --- | --- | --- |
| 0 | 0 | 0 | 0 |
| 1 | 0 | 0 | 0 |
| 0 | 1 | 0 | 0 |
| 0 | 0 | 1 | 0 |
| 0 | 0 | 0 | 1 |
| 1 | 1 | 0 | 0 |
| 1 | 0 | 1 | 0 |
| 1 | 0 | 0 | 1 |
| 0 | 1 | 1 | 0 |
| 0 | 1 | 0 | 1 |
| 0 | 0 | 1 | 1 |
| 1 | 1 | 1 | 0 |
| 1 | 1 | 0 | 1 |
| 1 | 0 | 1 | 1 |
| 0 | 1 | 1 | 1 |
| 1 | 1 | 1 | 1 |
| 4 | 1 | 0 | 0 |
| 4 | 0 | 1 | 0 |
| 4 | 0 | 0 | 1 |
| 1 | 4 | 0 | 0 |
| 1 | 0 | 4 | 0 |
| 1 | 0 | 0 | 4 |
| 0 | 4 | 1 | 0 |
| 0 | 4 | 0 | 1 |
| 0 | 0 | 4 | 1 |
| 0 | 1 | 4 | 0 |
| 0 | 1 | 0 | 4 |
| 0 | 0 | 1 | 4 |
| 3 | 2 | 0 | 0 |
| 3 | 0 | 2 | 0 |
| 3 | 0 | 0 | 2 |
| 2 | 3 | 0 | 0 |
| 2 | 0 | 3 | 0 |
| 2 | 0 | 0 | 3 |
| 0 | 3 | 2 | 0 |
| 0 | 3 | 0 | 2 |
| 0 | 0 | 3 | 2 |
| 0 | 2 | 3 | 0 |
| 0 | 2 | 0 | 3 |
| 0 | 0 | 2 | 3 |
| 3 | 7 | 0 | 0 |
| 3 | 0 | 7 | 0 |
| 3 | 0 | 0 | 7 |
| 7 | 3 | 0 | 0 |
| 7 | 0 | 3 | 0 |
| 7 | 0 | 0 | 3 |
| 0 | 3 | 7 | 0 |
| 0 | 3 | 0 | 7 |
| 0 | 0 | 3 | 7 |
| 0 | 7 | 3 | 0 |
| 0 | 7 | 0 | 3 |
| 0 | 0 | 7 | 3 |
| 5 | 1 | 1 | 0 |
| 5 | 1 | 0 | 1 |
| 5 | 0 | 1 | 1 |
| 0 | 5 | 1 | 1 |
| 1 | 5 | 1 | 0 |
| 1 | 5 | 0 | 1 |
| 1 | 0 | 5 | 1 |
| 0 | 1 | 5 | 1 |
| 1 | 1 | 5 | 0 |
| 1 | 1 | 0 | 5 |
| 1 | 0 | 1 | 5 |
| 0 | 1 | 1 | 5 |
| 1 | 3 | 3 | 0 |
| 1 | 3 | 0 | 3 |
| 1 | 0 | 3 | 3 |
| 0 | 1 | 3 | 3 |
| 3 | 1 | 3 | 0 |
| 3 | 1 | 0 | 3 |
| 3 | 0 | 1 | 3 |
| 0 | 3 | 1 | 3 |
| 3 | 3 | 1 | 0 |
| 3 | 3 | 0 | 1 |
| 3 | 0 | 3 | 1 |
| 0 | 3 | 3 | 1 |
| 1 | 2 | 3 | 0 |
| 1 | 3 | 2 | 0 |
| 2 | 3 | 1 | 0 |
| 2 | 1 | 3 | 0 |
| 3 | 1 | 2 | 0 |
| 3 | 2 | 1 | 0 |
| 1 | 2 | 0 | 3 |
| 1 | 3 | 0 | 2 |
| 2 | 1 | 0 | 3 |
| 2 | 3 | 0 | 1 |
| 3 | 1 | 0 | 2 |
| 3 | 2 | 0 | 1 |
| 1 | 0 | 2 | 3 |
| 1 | 0 | 3 | 2 |
| 2 | 0 | 1 | 3 |
| 2 | 0 | 3 | 1 |
| 3 | 0 | 1 | 2 |
| 3 | 0 | 2 | 1 |
| 0 | 1 | 2 | 3 |
| 0 | 1 | 3 | 2 |
| 0 | 2 | 1 | 3 |
| 0 | 2 | 3 | 1 |
| 0 | 3 | 1 | 2 |
| 0 | 3 | 2 | 1 |
| 7 | 1 | 1 | 1 |
| 1 | 7 | 1 | 1 |
| 1 | 1 | 7 | 1 |
| 1 | 1 | 1 | 7 |
| 4 | 4 | 1 | 1 |
| 4 | 1 | 4 | 1 |
| 4 | 1 | 1 | 4 |
| 1 | 4 | 4 | 1 |
| 1 | 4 | 1 | 4 |
| 1 | 1 | 4 | 4 |
| 3 | 3 | 3 | 1 |
| 3 | 1 | 3 | 3 |
| 3 | 3 | 1 | 3 |
| 1 | 3 | 3 | 3 |
| 1 | 2 | 3 | 4 |
| 1 | 2 | 4 | 3 |
| 1 | 3 | 2 | 4 |
| 1 | 3 | 4 | 2 |
| 1 | 4 | 2 | 3 |
| 1 | 4 | 3 | 2 |
| 2 | 1 | 3 | 4 |
| 2 | 1 | 4 | 3 |
| 2 | 3 | 1 | 4 |
| 2 | 3 | 4 | 1 |
| 2 | 4 | 1 | 3 |
| 2 | 4 | 3 | 1 |
| 3 | 2 | 1 | 4 |
| 3 | 2 | 4 | 1 |
| 3 | 1 | 2 | 4 |
| 3 | 1 | 4 | 2 |
| 3 | 4 | 2 | 1 |
| 3 | 4 | 1 | 2 |
| 4 | 2 | 3 | 1 |
| 4 | 2 | 1 | 3 |
| 4 | 3 | 2 | 1 |
| 4 | 3 | 1 | 2 |
| 4 | 1 | 2 | 3 |
| 4 | 1 | 3 | 2 |

The rows show the relative weighting vectors used; the first, second, third and fourth column corresponds to the ponderation of the “global”, “cytosol”, “mitochondrion” and “peroxisome” compartments, respectively.

Any$\vec{w}_{i}$ multiplied by a constant would give a vector that would generate a different objective function (a multiple of the original), with a different optimal (functional) value, but with the same optimal flux distribution when used as FBA objective. In the study carried out, the obtained flux distributions (when using every possible objective function in the FBA) are the important thing; for that reason and for simplicity, the relative weighting vectors are composed of integer numbers.
